# Evaluating the effectiveness and cost-effectiveness of the Smoking Treatment Optimisation in Pharmacies (STOP) intervention: protocol for a cluster randomised controlled trial

**DOI:** 10.1186/s13063-019-3368-6

**Published:** 2019-06-10

**Authors:** Ratna Sohanpal, Sandra Jumbe, Wai-Yee James, Liz Steed, Tammy Yau, Carol Rivas, Vichithranie Madurasinghe, Colin Houlihan, Vlad Berdunisov, Matthew Taylor, Stephanie J. C. Taylor, Chris Griffiths, Sandra Eldridge, Robert Walton

**Affiliations:** 1 Centre for Primary Care and Public Health, Blizard Institute, Barts and The London School of Medicine and Dentistry, Queen Mary University of London, London, UK; 2 California Northstate University, 9700 West Taron Drive, Elk Grove, CA 95757 USA; 3 Institute of Education, University College London, London, UK; 4 York Health Economics Consortium, University of York, York, UK

**Keywords:** Smoking cessation, Community pharmacy, Recruitment, Retention, Behaviour change, Cluster randomised controlled trial, Quit rate

## Abstract

**Background:**

NHS community pharmacies provide effective smoking cessation services; however, there is scope for increasing throughput and improving quit rates. This trial examines whether the Smoking Treatment Optimisation in Pharmacies (STOP) intervention can improve smoker engagement to increase service throughput, retention and quitting.

**Methods:**

This study is a pragmatic, cluster randomised controlled trial in 60 pharmacies in England and Wales. All workers in intervention pharmacies are offered STOP training while control pharmacies provide usual care. The STOP intervention, based on behavioural and organisational theories, comprises educational sessions for staff and environmental prompts in the pharmacy. Intervention fidelity is assessed by actors visiting pharmacies posing as smokers. The primary outcome is throughput, defined as the number of smokers who join the programme, set a firm quit date and undergo at least one stop smoking treatment session, and is measured using routinely collected data. Secondary outcomes include retention and quit rates at 4 weeks and continuous abstinence at 6 months verified by salivary cotinine. Cost-effectiveness is estimated using quality-adjusted life years and the probability that the intervention is effective at different levels of willingness to pay is calculated.

**Discussion:**

The trial will generate evidence to inform the public health smoking cessation strategy in England and Wales, and may help to shape service commissioning decisions. The STOP intervention model may help inform the undertaking of a range of health behaviour change tasks in community pharmacies.

**Trial registration:**

ClinicalTrials.gov, ISRCTN16351033. Retrospectively registered on 21 March 2017.

**Electronic supplementary material:**

The online version of this article (10.1186/s13063-019-3368-6) contains supplementary material, which is available to authorized users.

## Background

The tobacco epidemic is one of the biggest public health threats the world has ever faced, killing more than 7 million people each year [1]. Although the smoking prevalence has decreased recently in the United Kingdom, 19% of adults still smoke [2], which is a similar proportion to that observed in other developed countries [2]. A total of 1.7 million UK hospital admissions were recorded for conditions attributed to smoking in 2014/15 (22% more hospital admissions than in 2004/05). Thus, tobacco use has considerable negative implications for the individual, society and the National Health Service [2].

UK national guidance [3] stipulates that all health professionals should ask people if they smoke and give brief advice on smoking cessation, offering referral to the NHS Stop Smoking Service if appropriate. The Stop Smoking Service provides evidence-based treatment comprising intensive behavioural support (one-to-one or group), and discussion of medication options by personnel trained according to the National Centre for Smoking Cessation and Training (NCSCT) standard [4, 5].

A recent systematic review [6] suggested that community pharmacy-delivered interventions for smoking cessation (*n* = 14 studies) can be effective and cost-effective. However, the number of people using NHS Stop Smoking Services in community pharmacies has declined in recent years [6, 7] and there is evidence to suggest that participating pharmacies may target only smokers perceived as likely to quit [4, 7]. Retention of smokers within the service is poor, with about one-third of those setting a quit date being lost to follow up [4, 8].

There are no trials specifically focused on approaches to improve uptake and retention in the NHS community pharmacy smoking cessation service. Increasing throughput in this service may increase the numbers of people successfully giving up smoking, thus helping to reduce the burden of morbidity and mortality from tobacco use.

The protocol for this study has been written following the SPIRIT guidance [9] (see Additional file 1 for the populated SPIRIT checklist).

### Study aims

The primary aim of the Smoking Treatment Optimisation in Pharmacies (STOP) trial is to assess the effectiveness of a new training and service optimisation intervention (referred to as the STOP intervention) [10] on smoker throughput, retention and quit rates in the community pharmacy NHS Stop Smoking Programme.

The secondary aims are to assess cost-effectiveness and willingness to pay, smoker cessation, service user satisfaction with the stop smoking programme and pharmacy staff self-efficacy in delivery of stop smoking advice, and to conduct a process evaluation to evaluate fidelity of implementation, clarify causal mechanisms and identify factors associated with variation in outcomes [11].

### Intervention and comparator

#### Intervention group

The intervention community pharmacies are offered the STOP intervention, a service optimisation and behavioural skills training programme which is theory-driven and developed following the MRC recommendations for complex interventions [11] using a behaviour change framework [12] and embedded theory (social cognitive, self-determination) [13, 14]. We used a realist review of smoking cessation interventions in pharmacies [15] and diffusion of innovations theory [16] to plan optimal implementation.

Detailed descriptions of the development of the STOP intervention, including strategies to achieve the intended aims [4, 8, 10], acceptability and feasibility [17], and methods for assessing intervention fidelity are published elsewhere [18].

#### Control group

Community pharmacies in the control group continue with their usual practice; that is, delivery of the NHS Stop Smoking Programme according to the NCSCT programme [19, 20].

### Aims of the STOP intervention

The STOP intervention is targeted at all community pharmacy staff (including counter assistants as well as pharmacists) and aims to:

(i) increase throughput of smokers into the Stop Smoking Programme by building up communication skills and cessation knowledge of frontline community pharmacy staff (e.g. counter assistants); and
(ii) increase retention in the Programme and improve quit rates by building up consultation skills and knowledge about smoking cessation in stop smoking advisors.

## Methods

### Trial design

A pragmatic, cluster randomised controlled trial was performed with community pharmacies as the unit of randomisation. Pharmacies are allocated to the STOP training intervention or usual care (control group) using stratified randomisation with a 1:1 ratio.

**Fig. 1:**
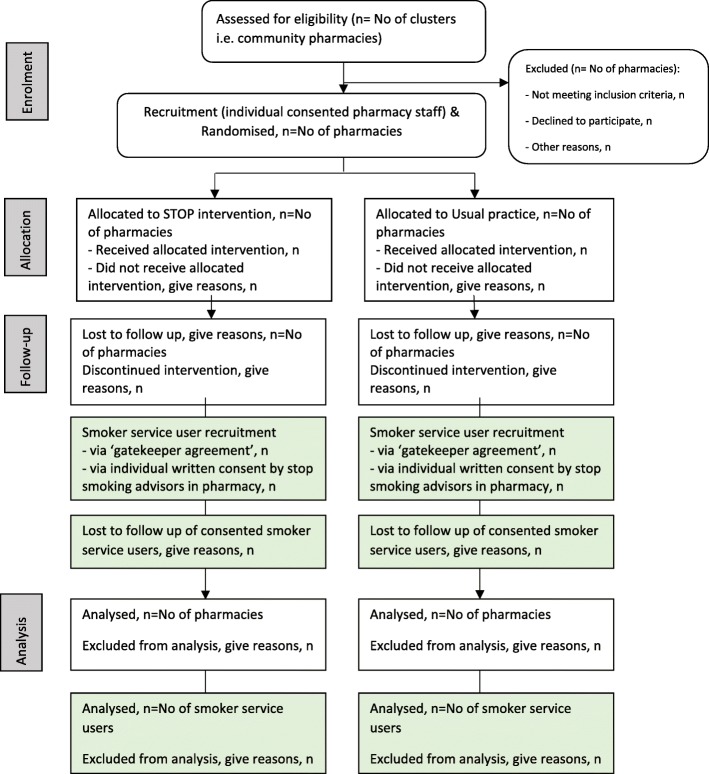
Flow diagram of progress of clusters and individuals through phases of the trial. STOP Smoking Treatment Optimisation in Pharmacies

The flowchart of the STOP trial is illustrated in Fig. 1.

**Fig. 2:**
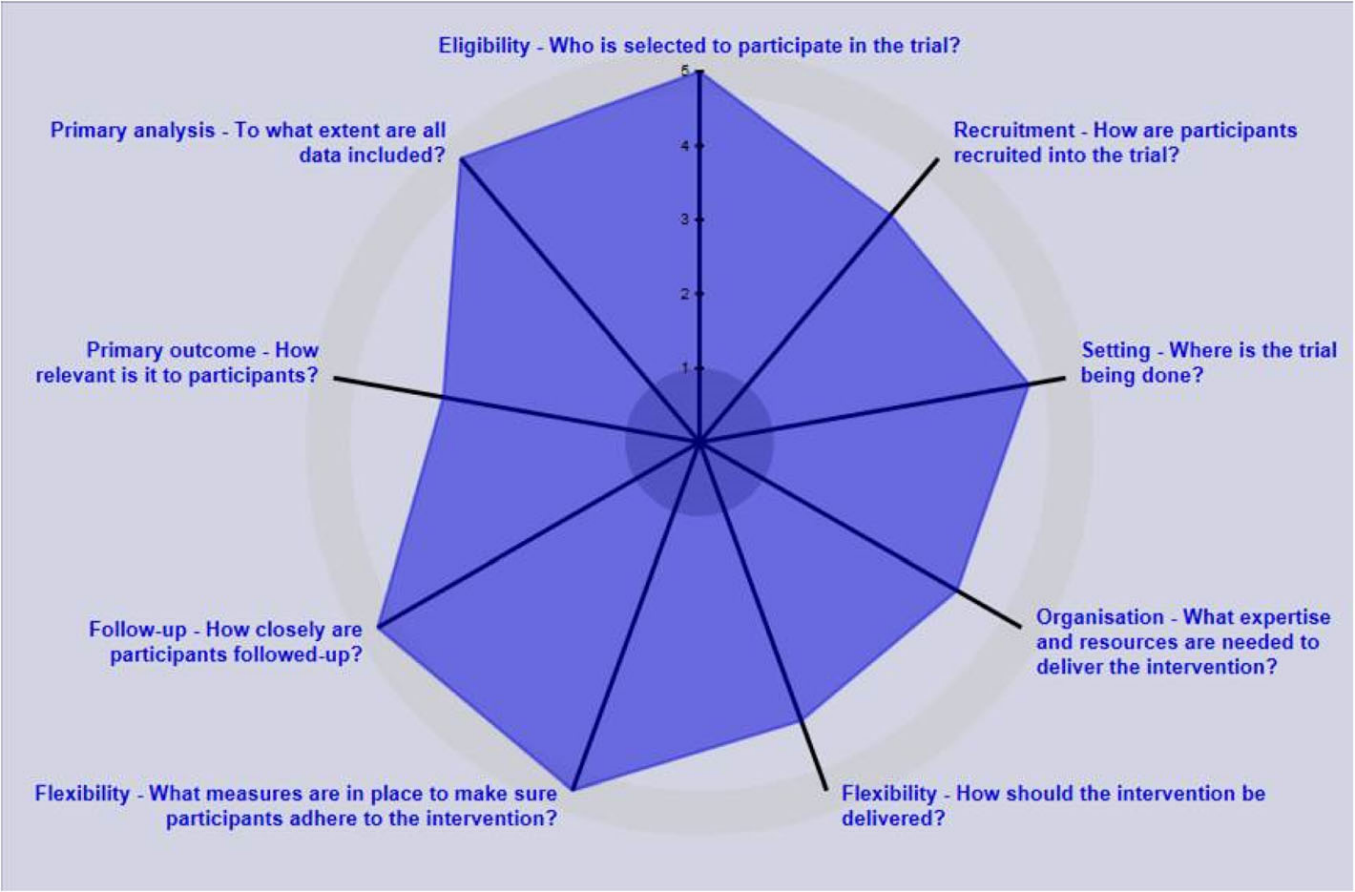
PRECIS-2 diagram illustrating that the trial lies towards the pragmatic end of the pragmatic–explanatory continuum. The Smoking Treatment Optimisation in Pharmacies trial was scored for each of nine domains from 1 = very explanatory to 5 = very pragmatic in group discussion with trialists and methodologists, and the results are displayed as a radar diagram [21]. PRECIS PRagmatic Explanatory Continuum Indicator Summary

Figure 2 shows a PRagmatic Explanatory Continuum Indicator Summary (PRECIS)-2 diagram [21] of the STOP trial at the smoker participant level. Each axis represents one of nine domains measuring how closely the trial is related to real life, scored from 1 = very explanatory to 5 = very pragmatic. Pragmatic trials are undertaken in the real world under usual care conditions and the results are intended to support a decision to deliver the intervention in routine practice [22]. The STOP trial is at the pragmatic end of the pragmatic–explanatory continuum.

### Setting

Sixty community pharmacies in London and Coventry, England and Cwn Taf, south east Wales.

### Sample size

The expected mean recruitment in a control cluster over the 11 months of the study is 165 (based on pilot/feasibility data) [17]. We consider that an increase of 33% in the intervention clusters to 220 would be sufficient to change commissioning policy. The standard deviation of throughput is expected to be approximately 63 in both groups, based on historical data from a service commissioner in the pilot/feasibility study and local authority data [23]. To have 90% power to detect this difference at the 5% significance level we need 56 clusters. We have added two clusters to each arm to allow for drop out, increasing the number of community pharmacies required to 60.

In order to assess quit rates, written individual consent is sought by advisors trained in the research processes (see Recruitment of smoker service users). Service users who join the Stop Smoking Programme over the continuous 11-month period are offered the opportunity to participate, with the aim of recruiting approximately 20 service users per pharmacy.

### Recruitment and randomisation of community pharmacies

#### Recruitment of community pharmacies and pharmacy workers

**Fig. 3:**
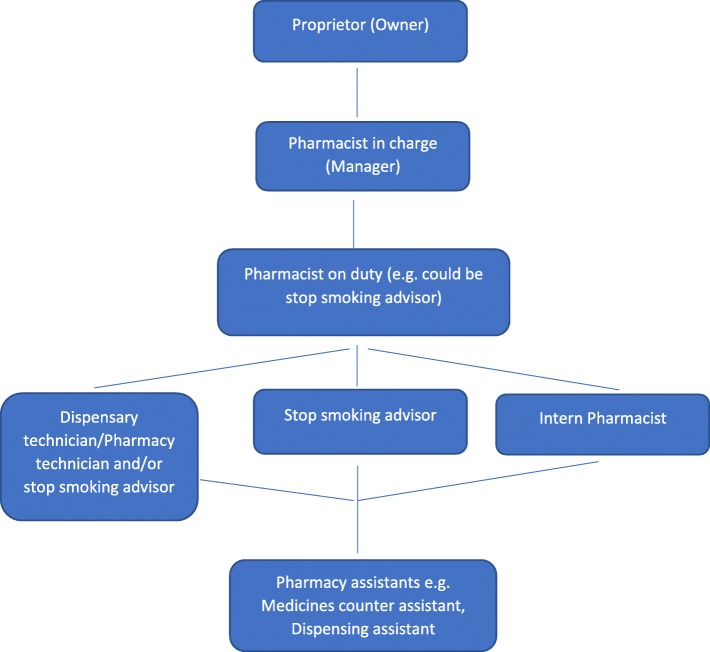
Illustration of the organisational structure of community pharmacies in England [24]. Proprietor (owner): if owns five or less community pharmacies, pharmacy is referred to as independent; if owns more than five community pharmacies, pharmacy is referred to as chain

Eligible community pharmacies are those providing the NHS Stop Smoking Programme and are identified from lists provided by service commissioners. Figure 3 gives an overview of the complex organisational structure of community pharmacies in England. The pharmacy owner is approached with an invitation letter or email, with an enclosed participant information sheet, and then followed up with a phone call after a few days. A meeting is then scheduled with the lead pharmacist and their staff (both stop smoking advisors and support or counter assistants) to discuss the study in detail and obtain written informed consent for participation. Following receipt of written consent, pharmacy characteristics (e.g. whether the pharmacy is a chain or independent) and pharmacy staff demographics are collected.

#### Randomisation

The community pharmacies are randomised to the STOP training intervention or to usual practice (no training) in a 1:1 ratio with stratified block randomisation for the pharmacy commissioner and number of prescriptions dispensed per month per pharmacy (as a proxy for pharmacy footfall), using an online system to ensure allocation concealment.

To reduce contamination due to staff crossover in small-chain pharmacies, a pharmacy chain where the owner has fewer than five pharmacies is randomised as one unit. For large pharmacy chains (owner has more than five pharmacies, e.g. Boots, Superdrug), individual outlets are randomised; each outlet is a separate randomising unit. The owners of large pharmacy chains or the head office confirmed to the study team at recruitment that there would be no staff crossover to the study team, and this was confirmed during trial monitoring visits.

The study team recruiting pharmacies emails an independent statistician with details of consented pharmacies for allocation. The independent statistician then performs the randomisation and notifies the study team of pharmacy allocation by email.

#### Blinding

The smoker service users are blind to the pharmacy’s allocation. Pharmacy staff are not blind to the intervention allocation; however, the collection of primary outcome data is via routine data collection, so the chances of bias are minimised. Follow-up assessments are conducted by a team not blind to the experimental intervention; however, validation of the reported smoking status is by salivary cotinine test performed by ABS Laboratories, which is blind to allocation.

### Participants

In this study there are two types of participants in relation to study outcomes and data collection (Table 2):

1. Pharmacy staff, including stop smoking advisors and support staff (related to process outcomes).
2. Service users in participating pharmacies (following ‘gatekeeper agreement’ [25]). The service users eligible for inclusion are self-reported current smokers aged 18 years and older who join the Stop Smoking Programme and attend the first stop smoking session (related to the primary outcome).

- A subgroup of service users who give individual, written, informed consent for additional data collection procedures (related to secondary outcomes).

### Recruitment of smoker service users

All service users who join the NHS Stop Smoking Service in participating pharmacies are considered part of the trial. Recruitment of service users, consenting for additional data collection, takes place over an 11-month period beginning immediately following the staff training session in intervention pharmacies and 1 month after the site initiation visit in control pharmacies (Table 2).

**Fig. 4:**
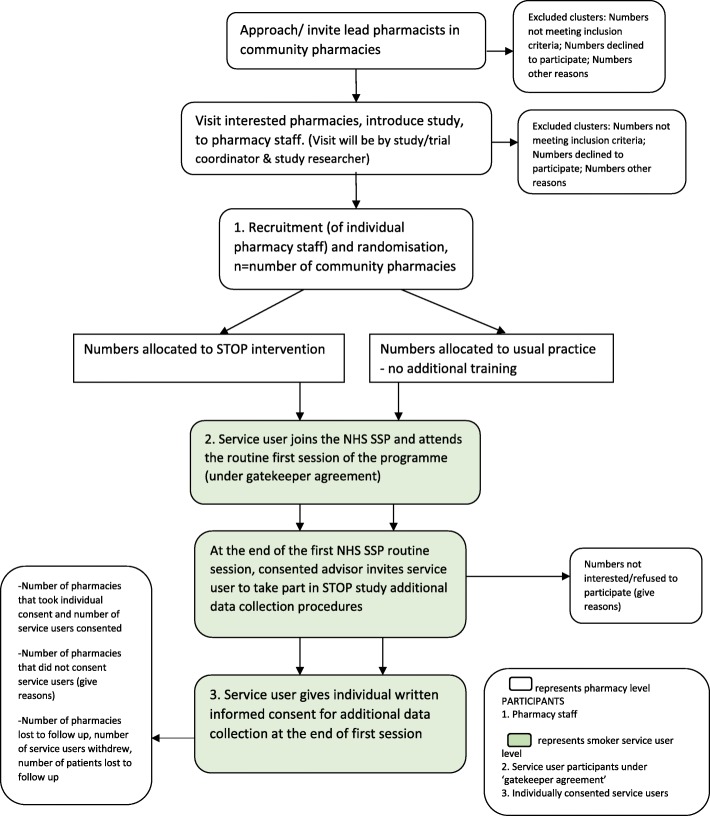
Process of recruitment. NHS SSP, National Health Service Stop Smoking Programme, STOP Smoking Treatment Optimisation in Pharmacies

Stop smoking advisors in all participating pharmacies undergo research-related training to take written informed consent and collect saliva samples from consenting service users. All service users who attend the first stop smoking session of the NHS Stop Smoking Programme (routine visit 1) are approached by their stop smoking advisor at the end of the session to participate in additional data collection procedures (Table 2) using a study information sheet and consent form. Written consent is taken from those interested within 24 h or at the next appointment in the pharmacy. This time period was found to be a convenient and acceptable way to consent service users in the feasibility study [17] and was approved by the ethics committee. Figure 4 illustrates the process of recruitment.

### Retention strategies

To maximise retention of pharmacies, pharmacy staff and service commissioners, we applied evidence-based strategies [26] and findings from our feasibility study [17].

A service contracting agreement is made with each participating pharmacy that includes the list/protocol of research-related work to be carried out. Training is provided in research-specific procedures and monitoring visits are planned to ensure the advisors carry out the research activities listed in the service contract. Good communication and relationships are maintained throughout between pharmacy staff and the study research team by regular contact using WhatsApp closed groups, email and phone calls. WhatsApp messages giving study information are sent to separately created control and intervention groups.

Pharmacy staff receive a £10 voucher for returning saliva samples to the study team and service users receive a £5 voucher for returning the satisfaction questionnaires. Service commissioners receive reimbursement for the time taken to send routine smoking cessation data to the study team at £40/h. STOP intervention training is incentivised by providing £40 in cash for counter assistants to attend one training session and £80 for pharmacists/stop smoking advisors to cover payment for attending two training sessions, including and travel expenses. Payments are based on hourly rates following discussion with the local Clinical Research Network (NOCLOR Research Support Service) that supports researchers in the set-up and conduct of research in primary care/community clinical settings. A ‘certificate of completion’ is given to each staff member for attending and completing the training.

### Data collection and measurements

#### Baseline characteristics

The pharmacy information and staff demographics are presented in Table 1, and service user demographics are presented in Table 2.

**Table 1:** Baseline demographics

	Baseline demographics		
Pharmacy information	Contractor code, type of pharmacy (independent/chain), total number of staff (both full-time and part-time), number of stop smoking advisors, number of pharmacy support staff. The study team also obtain the number of prescriptions dispensed monthly for each participating pharmacy from the Pharmacy and Appliance Contractor Dispensing data report (publicly available) [23]	At baseline	Directly from consented pharmacist/advisor
Pharmacy staff	Job title, highest education level, age, gender, smoking status	At baseline	Directly from consented pharmacist, advisor and support staff

**Table 2:** Smoking Treatment Optimisation in Pharmacies (STOP) trial data collection

Outcome/type of tissue sample	Description/process of data collection	Time of collection/follow-up point	Source of data
*STOP trial—outcome data collection*			
Throughput	From service commissioners	Received quarterly after STOP training intervention and study recruitment of service users	Routinely collected data under gatekeeper agreement (includes 12 months of historical pharmacy outcome data prior to randomisation date)
4-week retention rate	From service commissioners	Received quarterly after STOP training intervention and study recruitment of service users	Routinely collected data under gatekeeper agreement (includes 12 months of historical pharmacy outcome data prior to randomisation date)
4-week quit rate	From service commissioners	Received quarterly after STOP training intervention and study recruitment of service users	Routinely collected data under gatekeeper agreement (includes 12 months of historical pharmacy outcome data prior to randomisation date)
Continuous abstinence rate at 6 months	Consented participants who quit at 4 weeks are contacted at 6 months by a study researcher to ask: (i) their quit status—if response is yes (abstinent), a cotinine saliva collection kit is sent by post with instructions to give sample and return kit to ABS Laboratories in freepost envelope provided (ii) if participants completed the NCSCT questionnaire that was given to them at end of NHS SSP week 4—if response is no, the questionnaire is completed with researcher by telephone or sent by post with instructions to complete and return to study team in freepost envelope provided^a^	At 6 months if participant had quit at 4 weeks	Directly from individually consented service users for additional data collection
Detailed analysis on the effectiveness of the STOP training intervention	Data collected from STOP study participants comprise record number, exempt from prescription charge, gender, pregnant, age, occupation, ethnicity, national ethnicity, source of referral, referral by, patient practice, first session date, advisor name and nicotine replacement therapy products used. Data collected are anonymised. No personal details are recorded or used by the study team	At end of study	From individually consented service users for additional data collection (i.e. collected as routine data)
*STOP trial—process data collection*			
Satisfaction about the NHS SSP	NCSCT (short) satisfaction survey	Post 4 weeks of the NHS SSP	Directly from smoker service users (individually consented)
Self-efficacy in smoking cessation delivery	Assessing self-efficacy in delivery of smoking cessation using previously developed Likert scale [27]	At baseline and at 5 months from date of written consent	Directly from pharmacy staff
Study recruitment and retention rates. Reasons for non-participation and dropout	(i) Estimate study participation rate of (a) pharmacies (i.e. number of pharmacies recruited/total number approached to participate) and (b) pharmacy staff (i.e. number of pharmacy staff recruited/total number present in participating pharmacy and approached to participate) (ii) Estimate study retention rate of (a) pharmacies (i.e. number of pharmacies who dropped out/number recruited) and (b) pharmacy staff (i.e. number of pharmacy staff who dropped out/number recruited) (iii) Reasons for study non-participation and withdrawal among pharmacy staff		Recorded by study team
Intervention attendance and completion rates. Reasons for non-attendance and dropout	(i) Estimate STOP training intervention (a) attendance (i.e. number of staff who attended training/number assigned/who agreed to receive the intervention) and (b) dropout rates among pharmacy staff (i.e. number who dropped out of training/number attended) (ii) Reasons for non-attendance and non-completion		To be recorded by study team
Acceptability of intervention training and delivery in practice	Questionnaire using previously developed Likert scale [27]	At 5 months post written consent date	Directly from intervention pharmacy staff
Fidelity assessment at the pharmacy counter	Assessment of delivery of skills in practice at the pharmacy counter (in all 60 pharmacies) around engagement of service users into the NHS SSP via simulated clients using checklist developed for study	4–6 weeks post training	Directly from pharmacy staff
Fidelity assessment in pharmacy consultation room	Assessment of skills (from 33% of 30 intervention pharmacies, *n* = 10) around retention and quit success via audio-recording consultations	4–6 weeks post training	Directly from intervention audio-recordings of pharmacy staff
Views and experiences about the STOP training and its delivery in practice.	Interview data about views of the STOP training intervention and delivery of learnt skills in practice, reasons for non-attendance and non-completion	4 weeks post training	Directly from intervention pharmacy staff
Study recruitment and retention rates for additional data collection	(i) Estimate participation rate of smoker service users in the STOP study for additional data collection (i.e. total number of smokers who joined the NHS SSP and gave individual written consent/total number who joined the NHS SSP) (ii) Estimate service user retention rate in STOP study (i.e. number of consented service users who dropped out or are lost to follow up/total number of consented service users)		To be recorded by study team
Views and experiences about the NHS SSP with a focus on engagement and retention, reasons for completion and non-completion of the NHS SSP	Interview data about experience of the NHS SPP focusing on engagement and retention into the programme, reasons for non-completion of the programme	Post 4 weeks of the NHS SSP	Directly from service users
*STOP trial—tissue sample data collection*			
Saliva sample and DNA	Biochemical assessment of nicotine and DNA extraction and storage	At the end of the first session of the NHS SSP	Directly from individually consented smoker service users
Saliva sample	Cotinine assessment for continued abstinence	At 6 months among those who quit at 4 weeks of the NHS SSP	Directly from individually consented smoker service users

The focus of this study is only to collect the tissue samples and extract and store the DNA and assess salivary cotinine at 6 months to confirm cessation—critical to report for this study outcome

*NCSCT* National Centre for Smoking Cessation and Training, *NHS SSP* National Health Service Stop Smoking Programme

^a^A £10 voucher is sent in the same post to incentivise return of the questionnaire and saliva kit. Those not returning the questionnaire or kit are contacted a maximum of three times by the study team by telephone [26]

#### Primary outcome

The primary outcome measure is throughput assessed using routinely collected data supplied by service commissioners and defined as the number of smokers who:

1. join the stop smoking programme;
2. set a firm quit date; and
3. undergo at least one consultation on or prior to the quit date (i.e. a ‘treated smoker’ according to the Russell standard [28]).

#### Secondary outcomes

Using the Russell Standard criteria [28], we assess the following:

- The 4-week retention rate—the proportion of treated smokers retained at 4 weeks. A treated smoker is counted as ‘lost to follow up at 4-weeks’ (LFU4W) if, on attempting to determine the 4-week quit status, she/he cannot be contacted. The national guidance [29] recommends that pharmacy staff determine the quit status at 4 weeks in at least 85% of cases but there is no guidance given on the number of attempts allowed to follow up service users. Hence, the number of attempts and process of follow up used by staff may vary between pharmacies [17].
- The 4-week quit rate—the proportion of smokers who quit smoking at 4 weeks from the set quit date (i.e. a ‘carbon monoxide (CO)-verified 4-week quitter’).

In addition, continuous abstinence assessment of individually consented smokers—defined as the proportion of smokers who quit at 4 weeks (CO-verified) and remained so at 6 months (ascertained by telephone interview and verified by salivary cotinine)—is determined together with factors that may influence quit rates (e.g. gender, ethnicity).

Table 2 describes the type of data collected (i.e. trial outcome data, process data including intervention fidelity and tissue data), the source of data and the time point of collection.

**Fig. 5:**
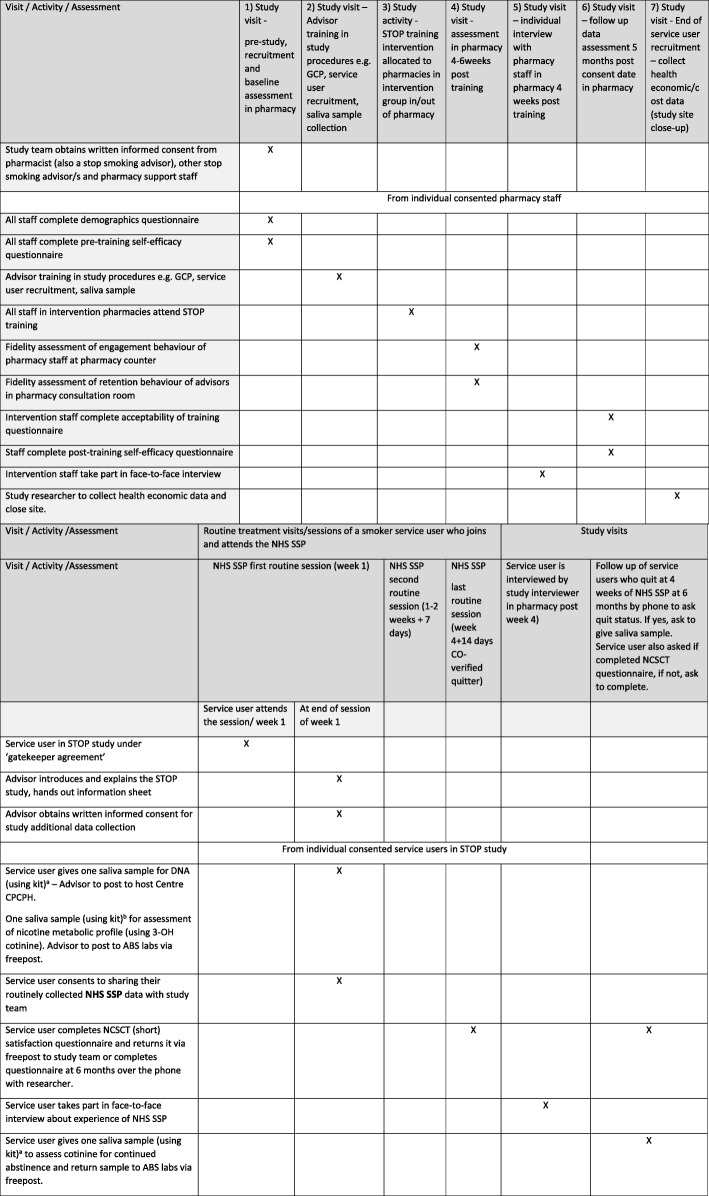
Standard Protocol Items: Recommendations for Interventional Trials (SPIRIT) figure. CO carbon monoxide, GCP Good Clinical Practice, NCSCT National Centre for Smoking Cessation and Training, NHS SSP, National Health Service Stop Smoking Programme, STOP Smoking Treatment Optimisation in Pharmacies

The SPIRIT figure (Fig. 5) provides information on the study visits and the activities/assessments at each visit.

### Economic evaluation

A cost-effectiveness analysis comparing the STOP intervention to usual care is conducted from the perspective of the NHS and personal social services. The cost of delivering the intervention to participants (e.g. training of pharmacy advisors and additional time spent consulting service users) is obtained by the study team and valued using published sources of UK costs [29, 30].

The intervention cost is combined with lifetime cost and quality-adjusted life years (QALYs) gained, estimated using a previously published economic model for analysis of smoking cessation and relapse [31]. A Markov cohort simulation predicts the likely smoking status and, subsequently, the health status of a cohort of 1000 hypothetical patients, each receiving a specific treatment strategy. The model accounts for various smoking-related morbidities, each associated with a cost and an impact upon the patient’s quality of life. Mortality rates are also included, for smokers and former smokers, and survival, combined with quality of life inputs, is used to generate expected QALYs.

The cost-effectiveness model uses the 6-month quit rate from the trial as the measure of short-term efficacy of the intervention. The long-term quit rate is unknown and is likely to be an important source of uncertainty, but is calculated using a combination of the 6-month quit rate, medium-term relapse rates and long-term ‘natural background’ quit rates, the latter two derived from the published literature. Background quit rates and the probability of relapse obtained from the literature simulate the proportion of the cohort who remain abstinent in future years.

One-way sensitivity analyses test the robustness of the model to assumptions of long-term effectiveness. A probabilistic sensitivity analysis gauges the combined effect of uncertainty around all model parameters simultaneously. The probability of cost-effectiveness of the intervention assuming different levels of willingness to pay per QALY is illustrated using a cost-effectiveness acceptability plane.

### Data management and analysis

Data are stored in the Queen Mary University of London (trial sponsor) secure virtualised environment with dual-factor authentication. A full statistical analysis plan is developed prior to release of allocation codes to anyone involved in writing that plan. We compare the numbers of participants enrolled in the Stop Smoking Programme between pharmacies allocated to the STOP training intervention and those who are not, using a mixed-effects Poisson regression model with the pharmacy as the random effect to allow for clustering. The dependent variable will be the number of ‘treated smokers’ from each pharmacy. The model will include two stratification factors—the commissioning site and the number of prescriptions/forms per month. Other covariates to be included in the model will be agreed a priori before the analysis plan is signed off.

## Discussion

This is the first randomised controlled trial to assess the effectiveness and cost-effectiveness of an intervention designed to optimise smoker throughput and retention in the NHS Stop Smoking Programme. The systematic, theory-based development and iterative refinement of the intervention before evaluation in the main trial is a strength of the study [10, 18].

As this is a multi-site trial in different parts of the United Kingdom, and is taking place in the setting where patients already receive their usual care, the likelihood that the findings can be generalised is increased. The intervention has potential to be modified to support other public health interventions with health behaviour or clinical outcomes delivered in community pharmacies.

The study is translational in nature with direct implications for health service provision in NHS community pharmacies and provides essential data to assess the cost-effectiveness of the intervention, thus informing commissioning decisions [32].

## Trial status

The trial is ongoing as protocol version 4, dated 21 March 2017. The recruitment period for pharmacies was from 1 June 2017 to 1 March 2018 and for smoker participants was from 1 June 2017 to 1 January 2019.

## Additional file

Additional file 1: SPIRIT 2013 checklist: recommended items to address in a clinical trial protocol and related documents (DOC 122 kb)
